# Developing evidence-based clinical imaging guidelines of justification for radiographic examination after dental implant installation

**DOI:** 10.1186/s12880-020-00501-3

**Published:** 2020-08-31

**Authors:** Min-Ji Kim, Sam-Sun Lee, Miyoung Choi, Hwan Seok Yong, Chena Lee, Jo-Eun Kim, Min-Suk Heo

**Affiliations:** 1 Department of Oral and Maxillofacial Radiology, Dental Research Institute, School of Dentistry, Seoul National University, 101 Daehak-ro, Jongno-gu, Seoul, 03080 Korea; 2 Division for Healthcare Technology Assessment Research, National Evidence-Based Healthcare Collaborating Agency, Seoul, Korea; 3 Department of Radiology, Korea University Guro Hospital, Seoul, Korea; 4 Department of Oral & Maxillofacial Radiology Yonsei University College of Dentistry, Seoul, Korea

**Keywords:** Imaging guideline, Cone Beam Computed Tomography (CBCT), Dental implant, Implant complication

## Abstract

**Background:**

This study aimed to develop evidence-based clinical imaging guidelines to assess the proper implant location following implant surgery and identify potential complications during follow-up.

**Methods:**

The guideline development process employed an adaptation methodology in accordance with the Korean clinical imaging guidelines (K-CIG). Core (Ovid-Medline, Ovid-Embase, National Guideline Clearinghouse, and Guideline International Network) and domestic databases (KoreaMed, KMbase, and KoMGI) were searched used to retrieve guidelines, and two reviewers analyzed the retrieved articles. The articles were included in this review using well-established inclusion criteria.

**Results:**

Our online search identified 66 articles, of which 3 were selected for the development of the guidelines. Consequently, based on these three guidelines, we formulated distinct recommendations regarding the appropriate imaging modalities that should be used following implant placement.

**Conclusions:**

Conventional imaging (e.g., periapical or panoramic radiography) should be the first choice for assessing the implant following its placement and osseointegration. The metal artifacts in Cone Beam Computed Tomography (CBCT) should be considered. However, CBCT is recommended for patients with sensory abnormalities following dental implant surgery to evaluate and identify the underlying cause of implant complications and to determine the appropriate treatment.

## Background

The use of dental implants has increased during the last few decades and has led to an increase in the number of related complications. Implant complications can be broadly divided as biological and technical. Biological complications include mucositis, peri-implantitis, and implant loss due to osseointegration failure, whereas technical complications involve fractures of implant prostheses, screw loosening, and loss of screw hole sealing [1]. In the event of a technical complication, the patient has the opportunity to notice the respective issue, and therefore treatment is feasible. In contrast, biological complications can be serious, including the need for removal of the implant fixture or development of severe mental damage stemming from irreversible nerve damage. Biological complications result in greater sequelae compared with technical complications.

Early diagnosis through periodic follow-up reduces the severity of complications and facilitate appropriate treatment [2]. Many of these complications are easily diagnosed on postsurgical imaging [3]. Radiological examinations for diagnosing the complications include panoramic radiography, periapical radiography, and cone-beam computed tomography (CBCT). These imaging modalities can help diagnosis of potential bone loss around the implant, osseointegration (implant-bone interface), and facilitate evaluation of the relationship between the surrounding anatomical structures and the implant [4–6].

Because no clear guideline on what type of modality is the first choice to assess this situation following dental implant surgery, this study aimed to develop evidence-based clinical imaging guidelines (CIGs) for this process.

## Methods

The guideline development process involved collaboration between The Korean Academy of Oral and Maxillofacial Radiology (KAMOFR) and the National Evidence based Healthcare Collaborating Agency (NECA) and Korean Society of Radiology [7, 8]. To develop such guidelines a development committee, a working group and a consensus group were formed.

### Committee composition

The development committee conducts a practical development process from selecting key questions to drawing final recommendations. The professional society recommends members who understand the development of clinical practice guidelines and who can actively participate in these process. A development committee is formed from these recommended members.

The working group consisted of nine oral and maxillofacial radiology experts and research methodology specialists who carried out actual tasks for development of clinical guidelines [7].

Finally, a consensus group consisted of seven nominated members from the five related societies who are expected to be end-users of clinical guidelines. They reviews key questions and participates in the expert panel survey (Delphi method) to agree on draft recommendations.

### Defining the key questions

The working group generated key questions and the development group and consensus group reviewed the generated key questions. The questions formed include the elements of PICO (Population/patient, Intervention/index, Comparator/control, Outcome) and are clearly stated.

The key questions were identified as follows

- KQ1. What is the appropriate imaging modality for follow-up after the dental implant surgery?
- KQ2. What is the appropriate imaging modality in patients with sensory abnormalities following dental implant surgery?

### Search for guidelines

Core databases such as Ovid-Medline, Ovid-Embase, Guideline International Network and National Guideline Clearinghouse were used to perform this guideline search. Moreover, three domestic research databases were investigated, including KoreaMed, KMbase, and KoMGI from 2000 until week one of May 2018. Extensive searches in databases used the keyword terms “*Dental implant,” “Radiograph,” “guideline,” “recommendation,”* and *“Cone-Beam Computed Tomography*.” When searching, we used operators so that there were no missing guidelines. The working group reviewed the search strategy and results and supplemented via manual search if any key guidelines were missing.

### Selection of searched guidelines

According to the selection criteria outline, two individuals in the working group independently reviewed literature. To ensure objectivity, a primary screening process a secondary selection process were both conducted. The primary screening involved the reviewing of the title and abstract of a study or guideline. In the secondary selection process, the full text of the literature was reviewed. Following these two processes, the working group selected the respective literatures and in cases where certain literatures had been excluded, the reason for exclusion was noted [7, 8].

The selection criteria were as follows: 1) PICOs included that match key questions, 2) clinical guidelines published in Korean or English, 3) clinical guidelines published since 2000.

The exclusion criteria were as follows: 1) patients in key questions were not targeted, 2) imaging modalities related key questions were not included, 3) appropriate results (diagnostic accuracy, efficacy, safety, prognostic impact and patient assessment, etc.) were not reported, 4) non-clinical guidelines, 5) no recommendation is given, 6) guidelines were not generated by the evidence based method based, 7) guidelines reported in languages other than English and Korean, 8) duplicated, 9) it is impossible to acquire the original text.

If there were disagreements between reviewers in the two selection processes, the clinical practice guidelines were selected through an agreement process.

### Search for recent literature

We investigated randomized controlled trials and observational studies whose literature has been reviewed since 2015.

### Quality assessment

The final selected guidelines underwent quality appraisal using the Korean Appraisal of guidelines for Research & Evaluation II tools [9]. Two appraisers independently selected from the development committee assessed the selected literature. Each evaluation category was assigned a score from 1 to 7 points. To ensure reproducibility and clarity, the reason for assigning these scores was noted. Differences in scores among the appraisers greater than four, led to the re-examination of the respective the literature. Essentially, guidelines scoring 50 or above in the “Rigor of development” domain, were considered as candidates for enrollment to develop K-CIGs [7].

### Grading the level of evidence and drafting the recommendation document

This process ensured that the guideline was up-to-date, acceptable, and applicable. Evidence tables which were consisted of the primary studies included in selected guidelines were made according to each key question, We extracted data from each primary studies on pre-defined format. Quality of studies was assessed by individual study level and included in evidence tables, This process was performed by two of authors independently then consensus reached. The level of evidence level in the K-CIG was merged with the evidence level in individual literature and it was categorized as high (I), moderate (II), low (III), or very low (IV) [7].

The draft recommendations consisted of recommendations for the key questions, a summary of the evidences, considerations, and references. Each recommendation includes the grade of the recommendation and the overall evidence level. The recommendation grade is composed of A, B, C, and I to indicate the direction of the recommendation, and the level of evidence indicates the strength of the recommendation [7].

### Agreement of the recommendation grade

The draft version of the recommendation produced by the working group was discussed the evidence level and grade of recommendation with the development committee. Ensuring agreement between the working group and development committees, allowed for the distinct determination of the validity of the recommendation document.

### Finalizing the recommendation document

The consensus group draws formal consensus through the Delphi method of anonymity. The first questionnaire is composed so that the key questions, draft recommendations, recommendation grades, and level of evidence can be viewed at a glance. The agreement level for each recommendation, the grade of each recommendation, and the evidence level were rated from strongly disagree (level 1) to strongly agree (level 9).

After the first survey, a second questionnaire is produced to reflect the degree of agreement and other opinions, and the survey is conducted. During the second survey, the distribution of all respondents and the evaluation results of each evaluator are provided by item, and the evaluator proceeds in a way that judges whether to correct or maintain the first evaluation result. Through this repetition round, consensus was reached.

## Results

### Pico

The PICO guideline was deduced based on key questions. Key questions were generated in the form of PICO questions by a working group. The PICO of key questions (KQ) 1 and 2 are as follows (Table 1).

**Table 1:** PICO of key questions

KQ	Population	Intervention	Comparators	Outcome
1	Patients with implants	CBCT	Panoramic and periapical radiographs	Diagnostic accuracy- alveolar bone height, osseointegration
2	Patients with sensory abnormalities following implant surgery	CBCT or CT (cross-sectional view)	Panoramic radiographs	Diagnostic possibility of nerve injury (inferior alveolar nerve)

### Search for guidelines

Tables 2 demonstrate the results acquired from domestic databases. No results were obtained from KGC and KoMGI. Tables 3, 4, 5 and 6 exhibit the acquired results from international databases. All searches were limited since the 2000.

**Table 2:** Search results from domestic databases

Searching date: 2018. 5.		
Search site	Search term	Searched literature
KoreaMed	Teeth [ALL] Implant [ALL] AND Guideline [ALL]	1
	Tooth [ALL] Implant [ALL] AND Guideline [ALL]	3
	“Dental Implant” [ALL] AND Guideline [ALL]	2
	Sum	6
	After omitting overlapped literatures	4
KMBASE	([ALL = Implant] AND [ALL = guideline])	5
	([ALL = Implant] AND [ALL = recommendation])	6
	([ALL = dental implant] AND [ALL = guideline])	15
	([ALL = dental implant] AND [ALL = recommendation])	5
	Sum	31
	After omitting overlapped literatures	25

**Table 3:** Search results from international databases: Ovid-Medline

Searching date: 2018. 5.			
	Number	Search term	Search result
P (Population)	1	exp Dental Implants/ OR ((tooth or teeth or dental) AND implant$).mp	40,053
I (Intervention)	2	exp Cone-Beam Computed Tomography/ OR CBCT.mp	7951
	3	exp Radiography, Dental/ OR (intraoral radiogra$ OR dental radiogra$).tw	20,874
	4	(imaging or radiolog$ or radiograp$).tw	843,422
	5	OR/2–4	856,832
P&C (Comparators)	6	1 AND 5	6156
Guideline filter	7	(guideline$ or recommendation$).ti. or (practice guideline or guideline).pt	99,097
Generalization	8	6 AND 7	18

**Table 4:** Search results from international databases: Ovid-Embase

Searching date: 2018. 5.			
	Number	Search term	Search result
P	1	‘tooth implant’/exp. OR ‘(tooth or teeth or dental) implant*’:ab,ti	8382
C	2	“cone-beam computed tomography”/exp. OR CBCT:ab,ti	15,233
	3	“tooth radiography”/exp. OR (“intraoral radiogra*” OR “dental radiogra*”):ab,ti	18,639
	4	(imaging or radiolog* or radiograp*):ab,ti	528,111
	5	OR/2–4	550,071
P&C	6	1 AND 5	1761
Guideline filter	7	guideline*:ti OR–recommendation*:ti	125,695
Generalization	8	6 AND 7	5

*: In Boolean search mode, use the asterisk wildcard character (*) to include alternative forms of words, plurals, etc

**Table 5:** Search results from international databases: GIN

Searching date: 2018. 5.	
Search term	Search result
Dental implant	0

**Table 6:** Searching result from international databases-NGC

Searching date: 2018. 5.	
Search term	Search result
Dental Implant	13

### Selection of searched guidelines

**Fig. 1:**
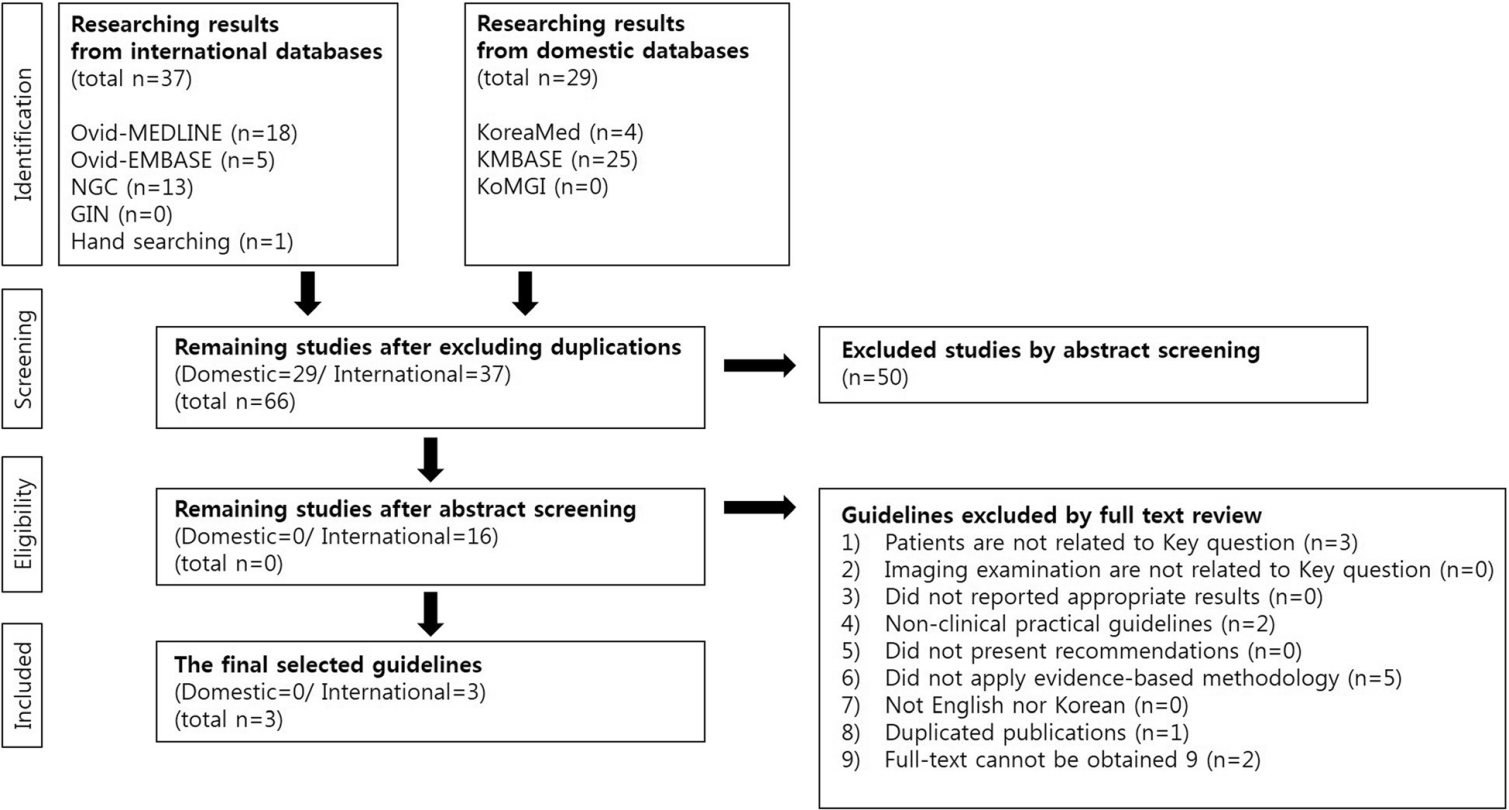
Selection process of searched guidelines

A total of 66 guidelines were retrieved from databases. Consequently, 50 guidelines were excluded following screening of their abstracts and 13 guidelines were excluded according to the selection and exclusion criteria (full-text review) Therefore, three guidelines were finally included in this review, as shown in Fig. 1.

### Search for recent studies

The latest studies were reviewed by 2015. Studies were selected provided that their evidence was related to the key questions were the latest or that can update the evidence table. Study designs corresponding to the top of the evidence pyramid (Meta analyses> systemic reviews> cohort studies>case-controlled studies) were primarily selected [10]. Consequently, six studies were included in this review.

### Quality assessment

Table 7 demonstrates the quality assessment of the three guidelines included in this review using the AGREEII instrument [9]. All of three guidelines received > 50 scores in the “Rigor of development” domain. Furthermore, Tables 8 and 9 show the recommendation matrix of the existing guidelines and the acceptability and applicability assessment of the three guidelines.

**Table 7:** Results of the quality assessment of the guidelines using AGREEII instrument

Title of guideline	AGREE score	Committee opinion
Guidelines for clinical use of CBCT: a review [11]	54	Recommended
E.A.O. guidelines for the use of diagnostic imaging in implant dentistry 2011. A consensus workshop organized by the European Association for Osseointegration at the Medical University of Warsaw [12]	79	Recommended
Radiation Protection No 172 CONE BEAM CT FOR DENTAL AND MAXILLOFACIAL RADIOLOGY [13]	90	Recommended

Not recommended: AGREE II < 50

**Table 8:** Recommendation matrix of the existing guideline

	Guideline A	Guideline B	Guideline C
Recommendation	Taking into account the justication principle, it was recommended that CBCT should be reserved as a supplementary imaging technique where conventional- radiography failed to answer the question for which imaging was required.	In most cases, conventional radiographs provide the necessary information, but additional images may be needed if there are complications after surgery (e.g., nerve damage or postoperative infections in relation to sinus cavities close to implants).	CBCT is not part of a “routine protocol” for postoperative examinations “unless there is a need for assessments in situations where some kind of complications have occurred, such as nerve damage, postoperative infections in relation to nasal and/or sinus cavities close to implants” (Harris et al. 2002).
Grading of recommendation	Not available	Not available	Not available

Guideline A; Guidelines for clinical use of CBCT: a review [11]

Guideline B: E.A.O. guidelines for the use of diagnostic imaging in implant dentistry 2011. A consensus workshop organized by the European Association for Osseointegration at the Medical University of Warsaw [12]

Guideline C: Radiation Protection No 172 CONE BEAM CT FOR DENTAL AND MAXILLOFACIAL RADIOLOGY [13]

**Table 9:** Acceptability and applicability assessment

	Acceptability and applicability			
	Evaluation list	Guideline A	Guideline B	Guideline C
Acceptability	Similarity of population	Yes	Yes	Yes
	Similarity of value and preference	Yes	Yes	Yes
	Similarity of benefit by recommendation	Yes	Yes	Yes
	Generally, acceptable	Yes	Yes	Yes
Applicability	Applicability of intervention/instrument	Yes	Yes	Yes
	Applicability of essential technique	Yes	Yes	Yes
	No legal and institutional barriers	Yes	Yes	Yes
	Generally, applicable	Yes	Yes	Yes

Guideline A; Guidelines for clinical use of CBCT: a review [11]

Guideline B: E.A.O. guidelines for the use of diagnostic imaging in implant dentistry 2011. A consensus workshop organized by the European Association for Osseointegration at the Medical University of Warsaw [12]

Guideline C: Radiation Protection No 172 CONE BEAM CT FOR DENTAL AND MAXILLOFACIAL RADIOLOGY [13]

### Grading the level of evidence and drafting the recommendation document

We compiled the individual literature related to the key question and prepared the evidence table by giving the level of evidence in our research (Table 10). Based on three guidelines, recommendations were then proposed. Each recommendation level and evidence level are as follows:

- *Recommendation 1. Panoramic and periapical radiography are recommended for the evaluation of implant position, osseointegration, and for following-up the bone level around the implant.*
  - *(Recommendation A, evidence level II).*
    - *Remark1. Conventional imaging (panoramic and periapical radiography) should be the first choice to perform these processes.*
    - *Remark2. CBCT imaging can be used on a limited basis to follow-up an implant placement due to the generation of dental implant and prostheses artifacts.*
- *Recommendation 2. CBCT is recommended for patients with sensory abnormality to evaluate the position of the implant and its surrounding structures, and to determine whether the implant is removed, following dental implant surgery.*
  - *(Recommendation B, evidence level II).*

**Table 10:** Evidence table

Author, year	Type of study	Patients (n)	Outcome	Note	Study quality (KCIG)
Mandelaris et al.2017 [14]	consensus statement		CBCT should be used as an adjunct to 2D dental radiology when, in the reasonable judgment of the clinician, the specific benefits to the patient as outlined above outweigh the risks.		2
Rios et al. 2017 [15]	evidence review	176 studies	Great heterogeneity still remains among the different available CBCT units, which is reflected in the wide range of effective CBCT doses estimated. The presence of inherent imaging artifacts caused by titanium implants significantly decreases the visualization of the bone implant interface in CBCT. It can cause significant interference when images are reviewed to assess implant placement and performance.		2
Yilmaz et al. 2017 [16]	survey	405 dentists	Given the serious nature of TG damage, dentists undertaking implant surgery should acquire knowledge and skills in the prevention, diagnosis, and management of dental implant–related TG nerve injury and receive specific training on justification and interpretation of CBCTs.		4
Bruschi et al.2017 [17]	consecutive patient	137 dental implants	Within the first year from implant placement, a bone loss resulted at a mean value of −1.11 ± 0.44 mm. After almost 3 years from implant placement, a mean bone gain of + 0.89 ± 0.39 mm was reported.	Reference Standard, Consecutive patient	2
Ter Gunne et al. 2016 [18]	RCT	40 patients	Mean radiographic marginal bone loss between baseline and the 3-year follow-up was 0.35 ± 0.63 mm for immediately loaded implants and 0.31 ± 0.96 mm for early loaded implants (*P* = .26).		2
Klokkevold. 2015 [19]	review	52 studies	Conventional imaging is the first choice standard for assessment and monitoring of bone levels around implants following placement and osseointegration. The use of CBCT imaging can help verify the implant position and facilitate the clinician’s decision making to remove or maintain an implant in a patient with postsurgical paresthesia.		4

### Finalizing the recommendation document

Delphi method is used, and following two rounds of assessment, the recommendation document was finalized. The average degree of agreement was 7.7 (standard deviation: 2.3) for recommendation 1 and 8.3 (standard deviation: 0.5) for recommendation 2, respectively (Table 11).

**Table 11:** Result of Delphi method

	Recommendation grade	Evidence level	Average	Minimum	Q1	Median	Q2	Maximum	SD	CV	Number of respondents
KQ1	A	II	7.7	3	8.0	8.5	9.0	9	2.3	0.3	7
KQ2	A	II	8.3	8	8.0	8.0	8.8	9	0.5	0.1	7

*Q* Quartile, *SD* standard deviation, *CV* coefficient of variation

## Discussion

A total of three guidelines were identified and which discussed the placement of implants. Literature evaluates the use of CBCT imaging in relation to implant surgery, and it is suggested that CBCT can be considered for use while reflecting the optimization of the image protocol. However, it should be used selectively as a subtest of two-dimensional image dentistry [15]. Similar to ionizing radiography, CBCT imaging should only be used in cases where the potential benefit to the patient outweighs the respective risks. The dentist should, therefore, consider this information, and hence assess whether the information obtained from the CBCT test can improve patient care, patient safety, and, ultimately, facilitates a more predictable and optimal treatment [15].

CBCT has a variety of application programs designed for the treatment of dental implants and should be used as an aid to 2-Dimentional radiographic images when clinicians believe that the benefits of this method outweigh the respective risks [12, 14].

Implants may generate artifacts that can cause significant interferences when inspecting the respective images for implant placement and performance [15].

Periapical radiography exhibited an average vertical bone gain of + 0.89 ± 0.39 mm at 36 months following implant placement, whereas bone loss averaged − 1.11 ± 0.44 mm in the first year of implant placement [17]. Three years and 1 month following implant placement, periapical radiography was performed using a film holder, and the mean radiographic marginal bone loss was 0.35 ± 0.63 mm for immediately loaded implants and 0.31 ± 0.96 mm for early loaded implants, respectively. The difference between the two groups was not statistically significant (*P* = 0.26) [18]. Several studies that investigated the correlation between height of implant abutment and peri-implant bone loss also used panoramic radiography and periapical radiography. And these studies showed significant results of the relationship between height of implant abutment and bone loss around the implant [20–22].

Conventional imaging (e.g., periapical or panoramic radiography) should the first selection criterion for the assessment and monitoring of bone levels around the implant following its placement and osseointegration. Implants and prostheses cause metal artifacts in CBCT, thus rendering this approach as difficult to perform accurate assessments with respect to implant-bone density and thickness. Therefore, CBCT should not be used for this purpose [23]. In the follow-up of implants, CBCT is often used as a means for early diagnosis of peri-implantitis. Due to metal artifact of the implant, the buccal bone of the implant is about 0.3 mm less (the diameter of the implant is 12–15% larger). Therefore, if the bone around the implant is not clearly visible due to the implant metal artifact in the cone beam CT image, the subjective judgment of the dentist is required for diagnosis [24].

Although the CBCT is useful to some extent in the structural analysis of the bony trabeculae, the CBCT is not reliable for evaluation of the bone density [25]. The Pseudo-Hounsfield value of CBCT is unreliable, so additional examination is necessary when CBCT is used in assessing quality and density of bone [26, 27]. These studies support the rationale that CBCT is not the primary examination in the follow-up after implant installation.

It is of great interest to have the capacity to increase the survivability of implants using proper occlusion adjustment and early treatment of peri-implantitis and by checking the implant location and surrounding bone mass during the follow-up period. Conventionally, radiation exposure and the significant economic burden are the limiting factors in this process, however, the radiation doses of the periapical and panoramic radiography are currently very low and the subsequent economic risk is also low compared to the alternative CT. More specifically, the radiation dose for each conventional radiography is 7.2 μSv for panoramic [23] and 1–8.3 μSv for periapical radiography, respectively [28]. In dental CBCT, the effective dose varies considerably among different apparatuses and can range from five to 1073 μSv. These variations stem from differences in the available fields of view, scanning times, and detector technologies [15].

Following implant surgery, CBCT is used to identify the location of the implant. In fact, CBCT can provide the operator with critical information of whether to remove or maintain implants in patients with paresthesia or anesthesia1 [12, 16]. A safety zone between the implant apex and the nerve canal is approximately 2 to 3 mm [29]. Direct mechanical injury to the nerve when drilling for implant placement or fixture can be very hazardous. Furthermore, if the apex of an implant is placed in the proximity of the nerve canal, it can cause compression-related injuries [30] that can facilitate the development of an altered sensation following implant placement. Consequently, this condition can be amended by immediate removal of the implant or by using pharmacologic treatments with high doses of NSAIDs to relieve the compression [30, 31].

The CBCT illustrates a three-dimensional relationship between implants and anatomical structures (inferior alveolar nerve and mental foramina) that are difficult to identify when using the panoramic image. Therefore, postoperative CBCT can be used to verify the location and distance between the implant fixture and the nerves, which is extremely useful in determining the treatment of sensory abnormalities [32].

Although this process can be harmful to the patient due to radiation exposure and economic burden involved, it is expected that it can alleviate the patients’ sensory abnormalities. Dentists performing implant surgery should then receive specific training to comprehend the optimal use of CBCT [19].

This guideline was created using systematic review and through end-user reviews and consensus. It will be very helpful when clinicians decide to use imaging to diagnose recall checks or neurological abnormalities after implant placement.

## Conclusion

This study developed evidence-based CIGs following implant placement.

The applicability and monitoring of these recommended guidelines need to be continuously assessed in the future to achieve optimal patient outcomes in clinical settings.

## Data Availability

Not applicable.

## Abbreviations

CBCT: Cone-beam computed tomography
K-CIG: Korean-Clinical imaging guidelines
CIG: Clinical imaging guidelines
KAMOFR: The Korean Academy of Oral and Maxillofacial Radiology
NECA: The National Evidence based Healthcare Collaborating Agency
PICO: Population/patient, Intervention/index test, comparator/control, and Outcome
KQ: Key questions

## References

[1] Adler L, Buhlin K, Jansson L (2020). Survival and complications: a 9- to 15-year retrospective follow-up of dental implant therapy. J Oral Rehabil.

[2] Hsu YT, Mason SA, Wang HL (2014). Biological implant complications and their management. J Int Acad Periodontol.

[3] Liaw K, Delfini RH, Abrahams JJ (2015). Dental implant complications. Semin Ultrasound CT MR.

[4] Kim DH, Ko MJ, Lee JH, Jeong SN (2018). A radiographic evaluation of graft height changes after maxillary sinus augmentation. J Periodontal Implant Sci.

[5] Rahman SA, Muhammad H, Haque S, Alam MK (2019). Periodic assessment of Peri-implant tissue changes: imperative for implant success. J Contemp Dent Pract.

[6] Schwindling FS, Hilgenfeld T, Weber D, Kosinski MA, Rammelsberg P, Tasaka A. In vitro diagnostic accuracy of low-dose CBCT for evaluation of peri-implant bone lesions. Clinical oral implants research. 2019;30(12):1200–8. 10.1111/clr.13533 31505065

[7] Choi SJ, Jeong WK, Jo AJ, Choi JA, Kim MJ, Lee M (2017). Methodology for developing evidence-based clinical imaging guidelines: joint recommendations by Korean Society of Radiology and National Evidence-Based Healthcare Collaborating Agency. Korean J Radiol.

[8] Kim MJ, Lee SS, Choi M, Ha EJ, Lee C, Kim JE (2020). Development of an evidence-based clinical imaging diagnostic guideline for implant planning: joint recommendations of the Korean academy of oral and maxillofacial radiology and National Evidence-based Healthcare Collaborating Agency. Imaging Sci Dent.

[9] Brouwers MC, Kho ME, Browman GP, Burgers JS, Cluzeau F, Feder G (2010). AGREE II: advancing guideline development, reporting and evaluation in health care. CMAJ..

[10] Martins RP, Buschang PH (2015). What is the level of evidence of what you are reading?. Dental Press J Orthod.

[11] Horner KOM, Taylor L, Glenny K, M A (2015). Guidelines for clinical use of CBCT: a review. Dento-Maxillo-Facial Radiol.

[12] Harris D, Horner K, Grondahl K, Jacobs R, Helmrot E, Benic GI (2012). E.A.O. guidelines for the use of diagnostic imaging in implant dentistry 2011. A consensus workshop organized by the European Association for Osseointegration at the Medical University of Warsaw. Clin Oral Implants Res.

[13] European Commission. Radiation protection No. 172: cone beam CT for dental and maxillofacial radiology (evidence based guidelines). Luxembourg: Directorate-General for Energy; 2012.

[14] Mandelaris GA, Scheyer ET, Evans M, Kim D, McAllister B, Nevins ML (2017). American Academy of periodontology best evidence consensus statement on selected Oral applications for cone-beam computed tomography. J Periodontol.

[15] Rios HF, Borgnakke WS, Benavides E (2017). The use of cone-beam computed tomography in management of patients requiring dental implants: an american academy of periodontology best evidence review. J Periodontol.

[16] Yilmaz Z, Ucer C, Scher E, Suzuki J, Renton T (2017). A survey of the opinion and experience of UK dentists: part 2: Risk assessment Strategies and the management of iatrogenic trigeminal nerve injuries related to dental implant surgery. Implant Dent.

[17] Bruschi GB, Cappare P, Bravi F, Grande N, Gherlone E, Gastaldi G (2017). Radiographic evaluation of Crestal bone level in Split-crest and immediate implant placement: minimum 5-year follow-up. Int J Oral Maxillofac Implants.

[18] Ter Gunne LP, Dikkes B, Wismeijer D, Hassan B (2016). Immediate and early loading of two-implant-supported mandibular overdentures: three-year report of loading results of a single-center prospective randomized controlled clinical trial. Int J Oral Maxillofac Implants.

[19] Klokkevold PR (2015). Cone beam computed tomography for the dental implant patient. J Calif Dental Assoc.

[20] Galindo-Moreno P, León-Cano A, Monje A, Ortega-Oller I, O'Valle F, Catena A (2016). Abutment height influences the effect of platform switching on peri-implant marginal bone loss. Clin Oral Implants Res.

[21] Lee BA, Kim BH, Kweon HHI, Kim YT (2018). The prosthetic abutment height can affect marginal bone loss around dental implants. Clin Implant Dent Relat Res.

[22] Vervaeke S, Dierens M, Besseler J, De Bruyn H (2014). The influence of initial soft tissue thickness on peri-implant bone remodeling. Clin Implant Dent Relat Res.

[23] Lee C, Lee SS, Kim JE, Symkhampha K, Lee WJ, Huh KH (2016). A dose monitoring system for dental radiography. Imaging Sci Dent..

[24] Vanderstuyft T, Tarce M, Sanaan B, Jacobs R, de Faria Vasconcelos K, Quirynen M (2019). Inaccuracy of buccal bone thickness estimation on cone-beam CT due to implant blooming: an ex-vivo study. J Clin Periodontol.

[25] Corpas Ldos S, Jacobs R, Quirynen M, Huang Y, Naert I, Duyck J (2011). Peri-implant bone tissue assessment by comparing the outcome of intra-oral radiograph and cone beam computed tomography analyses to the histological standard. Clin Oral Implants Res.

[26] Pauwels R, Jacobs R, Singer SR, Mupparapu M (2015). CBCT-based bone quality assessment: are Hounsfield units applicable?. Dentomaxillofac Radiol.

[27] Pauwels R, Nackaerts O, Bellaiche N, Stamatakis H, Tsiklakis K, Walker A (2013). Variability of dental cone beam CT grey values for density estimations. Br J Radiol.

[28] Gijbels F, Jacobs R, Sanderink G, De Smet E, Nowak B, Van Dam J (2002). A comparison of the effective dose from scanography with periapical radiography. Dentomaxillofac Radiol..

[29] Misch CE, Crawford EA (1990). Predictable mandibular nerve location--a clinical zone of safety. Int J Oral Implantol.

[30] Khawaja N, Renton T (2009). Case studies on implant removal influencing the resolution of inferior alveolar nerve injury. Br Dent J.

[31] Al-Ouf K, Salti L (2011). Postinsertion pain in region of mandibular dental implants: a case report. Implant Dent.

[32] Al-Sabbagh M, Okeson JP, Bertoli E, Medynski DC, Khalaf MW (2015). Persistent pain and neurosensory disturbance after dental implant surgery: prevention and treatment. Dent Clin North Am.

